# Evaluating the effectiveness of precision integrative nursing plans in nursing quality management at a digestive endoscopy center

**DOI:** 10.12669/pjms.42.4.13594

**Published:** 2026-04

**Authors:** Na Zhao, Fengjuan Wang

**Affiliations:** 1 Na Zhao Endoscopic Diagnostic and Treatment Centre, Affiliated Hospital of Hebei University, Baoding 071000, Heibei, China; 2 Fengjuan Wang Sterilization Supply Centre, Affiliated Hospital of Hebei University, Baoding 071000, Heibei, China

**Keywords:** Digestive Endoscopy, Nursing Quality, Precision Integrative Nursing, Patient Satisfaction

## Abstract

**Objective:**

To evaluate the effects of precision integrative nursing plans in nursing quality management at a digestive endoscopy centre.

**Methodology:**

This retrospective study involved three hundred patients diagnosed and treated at the digestive endoscopy centre of the Affiliated Hospital of Hebei University from January 2024 to June 2025. Participants were randomly assigned by a number table to the control group (n= 150) or the observation group (n= 150), which received conventional nursing and precision integrative nursing, respectively. The two groups were compared in terms of nursing quality, health knowledge scores, nursing satisfaction and complication incidence.

**Results:**

The observation group achieved significantly higher results than the control group for endoscope cleaning and disinfection, instrument management, environmental management and intestinal-preparation compliance rates (*P*< 0.05). Patients and family members in the observation group answered at least three questions correctly, achieving health knowledge scores of 90.67% and 86.67%, respectively, and exceeding those of the control group (*P*< 0.001). Satisfaction levels were also greater in the observation group than in the control group across three dimensions: treatment environment, service attitude and nursing skills (*P*< 0.001). Additionally, only five patients in the observation group developed complications, representing an incidence rate of 3.33%, lower than 10.00% in the control group (15/150; *P*< 0.05).

**Conclusion:**

Implementing precision integrative nursing plans in nursing quality management at the digestive endoscopy centre significantly improves nursing quality indicators, increases the health knowledge of patients and their families and raises patient satisfaction while reducing complication incidence.

## INTRODUCTION

In recent years, the use of digestive endoscopy has expanded with technological advances. Procedures now range from routine gastroscopy and colonoscopy to complex therapies such as endoscopic mucosal resection and endoscopic submucosal dissection, leading to a substantial increase in the number of patients undergoing examinations and treatments.^1^-^4^ Therefore, the digestive endoscopy centre must manage high patient volumes, making improvements in patient experience and nursing quality a priority for nursing management. Specifically, the precision integrative nursing plan is patient-centred and builds a full-cycle management model through standardised processes, personalised assessment and multidisciplinary collaboration, aiming to provide more precise and high-quality nursing services to patients.^5^-^7^ On this basis, this study systematically evaluated the effectiveness of precision integrative nursing plans across four dimensions: nursing quality, health knowledge, patient experience and complication prevention and control.

## METHODOLOGY

This retrospective study included three hundred patients diagnosed and treated at the digestive endoscopy centre of the Affiliated Hospital of Hebei University from January 2024 to June 2025. Patients were allocated by a random-number table to the control group (n= 150) and the observation group(n= 150), which received conventional nursing and precision integrative nursing plans, respectively. After collecting and organizing the cases, used the sealed envelope system.

### Ethical approval:

The study was approved by the Institutional Ethics Committee of Affiliated Hospital of Hebei University (HDFYLL-KY-2024-105; May 6, 2024), and written informed consent was obtained from all participants.

### Inclusion criteria:

- Provided informed consent.
- No communication barriers.
- Age ≥18 years.

### Exclusion criteria:

- Severe mental disorders.
- Previous digestive endoscopy-related examination or treatment at the Affiliated Hospital of Hebei University.

The control group received conventional nursing. The observation group received precision integrative nursing. The evaluation process of patients’ outcomes was double-blind.−

### Team Establishment:

A six-member nursing team was formed: one nurse-in-charge (team leader), three senior nurses and two nurses. The team leader oversaw training, assessment scoring and multidisciplinary coordination. Senior nurses managed high-risk patients and handled in-procedure emergencies. Nurses conducted preoperative education, prepared instruments and performed basic nursing operations.

### Preoperative Precision Assessment and Preparation:

Patients were stratified by age (≥65 years or not), comorbidities (e.g. diabetes, coagulation disorders, etc.) and examination type (including complex procedures like endoscopic submucosal dissection).^8^ A risk classification table categorised patients as high (all three items), medium (two items) or low risk (one item). Health education was tailored to risk level: for medium and low-risk patients, materials on endoscopy precautions were provided, with key information highlighted using a fluorescent marker. Fasting and laxative administration methods were emphasised. For high-risk patients, one-to-one demonstrations were conducted using models while indicating potential discomfort points and corresponding measures. After health education, a question-and-answer (Q&A) session was held; incorrect answers were re-explained and flagged for follow-up.

### Intestinal preparation:

Laxative dosage was adjusted according to constipation history: no constipation: oral polyethene glycol; mild constipation: polyethene glycol + lactulose; severe constipation: sodium phosphate + enema.^9^-^11^ One day before the examination, a phone call was made to confirm the preparation status and assess stool characteristics. If criteria were unmet, remedial measures were initiated, such as administering an additional 200 mL of polyethene glycol.

### Environmental management:

The endoscopy room was kept clean and quiet. Bed surfaces and related equipment were wiped and disinfected daily with a chlorine-containing disinfectant. Temperature was maintained at 22-25°C with humidity at 50%-60%, with weekly air disinfection. Standardised instrument packs were assembled by examination type (gastroscopy, colonoscopy or endoscopic ultrasound) and labelled with disinfection validity and usage order to reduce preparation time and improve efficiency. For single-use instruments, expiry dates and packaging integrity were checked; for reusable instruments, cleaning, disinfection and sterilisation followed regulations. Moreover, an instrument management ledger recorded entry and exit status, usage frequency and maintenance times.

### Intraoperative Precision Coordination and Operation:

Proper lighting avoided direct strong light on the patient’s eyes in the endoscopy examination room, and ventilation was maintained. Instruments were used under sterile operation principles to avoid cross-infection, and preliminary processing was performed promptly after use, with instruments placed in designated recycling containers. Operations strictly followed the ‘Technical Specifications for Endoscope Cleaning and Disinfection’, with bedside pre-treatment provided immediately after endoscope use.^12^,^13^ Heart rate, blood oxygen saturation, blood pressure and other data were continuously monitored. When abnormalities occurred, measures were taken, such as adjusting oxygen flow and rousing the patient if oxygen saturation dropped below 90%. During examination, disposable sterile drapes covered non-examined areas to protect the patient’s privacy. During colonoscopy, the examination table was tilted by 15° for thin patients and 25°for obese patients. For gastroscopy, the patient was positioned left lateral with the table tilted at 10°.

### Postoperative Precision Management and Follow-up:

In the recovery room, patients were observed for 30 minutes. After confirmation of no dizziness or abdominal pain, patients were discharged along with explanations and postoperative care instructions. For high-risk patients, observation time was extended to 1 h with ECG monitoring. A nurse accompanied patients back to the ward and conducted ‘three confirmations for bedside handover’ (consciousness, tubes and skin) with the responsible doctor. Dietary advice was tailored to examination type, with a gradual transition from liquid to normal diet after gastroscopy.

### Multidisciplinary Collaboration:

The endoscopy, anaesthesiology, infection control and information departments were integrated into a unified nursing platform for resource sharing and process coordination. For example, the endoscopy nurse notified the anaesthesiology department and the responsible doctor promptly in case of abnormal patient conditions to ensure a coordinated response.

### Observation indicators:

- ***Nursing Quality. Endoscope Cleaning and Disinfection:*** The team leader assessed compliance with bedside pre-treatment, standardised cleaning and disinfection steps, appropriate disinfectant concentrations and contact times and correct storage conditions. A case was recorded as qualified only when all criteria were met. Instrument Management Quality: The team leader verified that all required instruments were prepared in advance and that instrument packs were accurate. Environmental Management Quality: The team leader confirmed that the temperature and humidity of the endoscopy examination room met the requirements. Intestinal-Preparation Compliance Rate: Compliance was determined from stool characteristics during the pre-examination phone follow-up. Cleanliness with no faecal residue and clear liquid output indicated compliance.
- ***Health Education Effectiveness:*** After preoperative health education, the team leader assessed patients and their families in a Q&A format with five targeted questions per patient. A score of four or more correct answers indicated a good understanding of the health education content.
- ***Patient Satisfaction:*** After the observation period, patients rated their satisfaction with the nursing service based on their experiences on a 10-point scale. It covered treatment environment, service attitude and nursing skills. Higher scores indicated better satisfaction.
- ***Safety Assurance Dimension:*** Patients’ medical records, nursing documentation and related records were reviewed to count complications, such as bleeding, perforation or anaesthetic reactions, which occurred during digestive endoscopy examinations or treatments.

### Statistical analysis:

SPSS 26.0 was used for data processing. Categorical data (gender, education level, nursing quality, health knowledge and complications) were expressed as n (%) and analysed with the *χ^2^* test. Continuous data (age, number of correct questions and satisfaction scores) were expressed as (*x̅*+*s*) and analysed with the independent samples t-test. *P* < 0.05 was considered statistically significant.

## RESULTS

**Fig.1:**
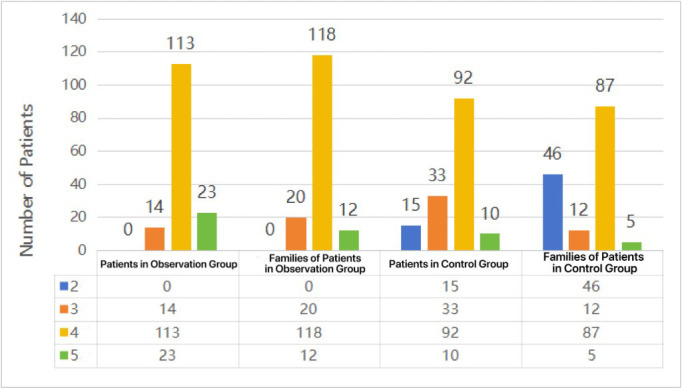
Correct Answers by Patients and Their Family Members in the Two Groups.

Statistical analysis showed no statistical differences in baseline data between the two groups (Table-I). The observation group outperformed the control group in endoscope cleaning and disinfection, instrument management, environmental management and intestinal-preparation compliance rate (*P* < 0.05; Table-II). Patients and family members in the observation group answered at least three questions correctly, achieving health knowledge scores higher than those in the control group (*P* < 0.001; Table-III; Fig.1).

**Table-I:** Comparison of Baseline Data between the Two Groups (*x̅*+*s*)/ [n (%)].

Item	Group	Observation Group (n = 150)	Control Group (n = 150)	χ^2^/t value	P value
Age (Years)		48.41 ± 7.76	47.69 ± 7.49	0.818	0.414
Gender	Male	79 (52.67)	85 (56.67)	0.484	0.487
	Female	71 (47.33)	65 (43.33)		
Education Level	Middle school or below	38 (25.33)	29 (19.33)	1.596	0.450
	High school and junior college	69 (46.00)	73 (48.67)		
	Undergraduate or above	43 (28.67)	48 (32.00)		

**Table-II:** Comparison of Nursing Quality between the Two Groups [n (%)].

	Endoscope Cleaning and Disinfection	Instrument Management	Environmental Management	Intestinal-Preparation Compliance Rate
Observation Group (n = 150)	141 (94.00)	139 (92.67)	140 (93.33)	143 (95.33)
Control Group (n = 150)	128 (85.33)	124 (82.67)	121 (80.67)	130 (86.67)
*t* value	6.080	6.937	10.640	6.878
*P* value	0.014	0.008	0.001	0.009

**Table-III:** Comparison of Health Education Knowledge between the Two groups (*x̅*+*s*)/[n (%)].

	Correct answer by patients (n)	Knowledge of patients	Correct answer by patients’ families (n)	Knowledge of patients’ families
Observation Group (n = 150)	4.06 ± 0.49	136 (90.67)	3.95 ± 0.46	130 (86.67)
Control Group (n = 150)	3.65 ± 0.75	102 (68.00)	3.34 ± 0.95	92 (61.33)
*t* value	5.624	23.502	7.013	25.017
*P* value	<0.001	<0.001	<0.001	<0.001

Satisfaction scores were higher in the observation group than those of the control group in three aspects: treatment environment, service attitude and nursing skills (*P* < 0.001; Table-IV). Complications were fewer in the observation group: five cases (3.33%) versus 10.00% (15/150) in the control group (*P* < 0.05; Table-V).

**Table-IV:** Comparison of Patient Satisfaction between the Two Groups (*x̅*+*s*, points).

	Treatment Environment	Service Attitude	Nursing Skills
Observation Group (n = 150)	7.56 ± 0.94	8.59 ± 0.68	8.17 ± 0.87
Control Group (n = 150)	6.23 ± 1.13	7.41 ± 0.84	7.12 ± 1.03
*t* value	11.121	13.429	9.595
*P* value	<0.001	<0.001	<0.001

**Table-V:** Comparison of Complications between the Two Groups [n (%)].

	Incidence	Non-incidence
Observation Group (n = 150)	5 (3.33)	145 (96.67)
Control Group (n = 150)	15 (10.00)	135 (90.00)
*χ^2^* value	5.357	
*P* value	0.021	

## DISCUSSION

Data analysis showed better performance in the observation group for endoscope cleaning and disinfection, instrument management, environmental management and intestinal-preparation compliance rate. Standardised processes make nursing quality controllable by reducing operational randomness and lowering nursing error rates.^14^ Strict adherence to cleaning and disinfection procedures increases compliance because each step is executed accurately. Instrument management improved when maintenance, calibration and detailed record-keeping are routine, keeping devices in optimal condition. Environmental management benefits from clear zoning and well-defined requirements, which support a comfortable and hygienic treatment setting. A higher intestinal-preparation compliance rate reflects precise nursing tailored to patient differences. For instance, polyethene glycol is selected appropriately, and preparation plans are personalised for patients with constipation, thus enhancing the effectiveness of intestinal preparation.^15^,^16^ During endoscopic examination, patients are prone to fear, and improvements in nursing quality have not been a clinical focus. Therefore, effective nursing solutions remain a research priority.^17^ As an innovative model to optimise care, the precision integrative nursing plan was evaluated to provide evidence for improving nursing services at the digestive endoscopy centre and raising patient satisfaction.

Precision nursing also influences health education and patient satisfaction. By marking key points and delivering risk-stratified education, patients and family members in the observation group obtained higher health knowledge than those in the control group. The approach emphasises comprehensive assessment to accurately identify risk levels. For high-risk patients, detailed explanations of disease-related knowledge, treatment precautions and possible complications strengthen self-management. For low-risk patients, basic health knowledge is disseminated in an accessible form to meet their diverse needs.

The satisfaction levels of the observation group outperformed those of the control group in the treatment environment, service attitude and nursing skills. Improvements in environmental management directly increased satisfaction with the treatment environment. Regarding service attitude, nursing staff provide tailored measures based on patients’ conditions, making patients feel specially attended to and that the treatment is targeted. For nursing skills, precise implementation of procedures according to patient characteristics, such as adjusting the bed tilt by body type, can enhance nursing effectiveness and reduce discomfort. Collectively, these factors explain the superior satisfaction ratings in the observation group. Consistent with these findings, other researchers 18,19 have reported that precision nursing can enhance nursing satisfaction in urological surgery and pulmonary nodule treatment.

Regarding complications, the observation group had a lower overall incidence. Precision nursing requires close monitoring of vital signs (e.g. blood pressure, heart rate and blood oxygen saturation) and ongoing assessment of symptoms, allowing subtle changes in patients’ conditions to be detected in real time. When abnormalities are identified, effective intervention measures are implemented in a timely manner to prevent complications at an early stage.^20^

### Limitations:

This study included a relatively small sample and did not conduct long-term follow-up. Future research should use larger samples and extend follow-up to further validate the findings of this study. We will continue to do large-scale sample and long-term follow-up studies to improve the credibility of the conclusion and the level of evidence.

## CONCLUSIONS

Implementing precision integrative nursing plans in nursing quality management at the digestive endoscopy centre shows marked effectiveness. Compared with conventional nursing, this approach not only improves compliance with quality indicators for tasks such as endoscope cleaning and disinfection but also effectively increases the health knowledge of patients and their family members; raises satisfaction with the treatment environment, service attitude and nursing skills; and reduces complication incidence. Precision integrative nursing plans demonstrate high clinical application value.

### Authors’ Contributions:

**NZ:** Designed and did statistical analysis & editing of manuscript, is responsible for integrity of research.

**FW:** Conceived designed, Literature search, Did data collection and manuscript writing.

All authors have read and approved the final manuscript.
